# *Tropheryma whipplei* aortic valve endocarditis, cured without surgical treatment

**DOI:** 10.1186/1756-0500-5-600

**Published:** 2012-10-30

**Authors:** Ahmet Algin, Marjolijn Wegdam-Blans, Kees Verduin, Hans Janssen, Jan-Melle van Dantzig

**Affiliations:** 1 Department of Cardiology, Catharina hospital Eindhoven, Michelangelolaan 2, Eindhoven, 5623EJ, the Netherlands; 2 Department of Microbiology, Catharina hospital Eindhoven, Michelangelolaan 2, Eindhoven, 5623EJ, the Netherlands; 3 Department of Microbiology, Sint Anna hospital Geldrop, Bogardeind 2, Geldrop, 5664EH, the Netherlands; 4 Department of Cardiology, Sint Anna hospital Geldrop, Bogardeind 2, Geldrop, 5664EH, the Netherlands

**Keywords:** Endocarditis, *Tropheryma whipplei*, Aortic valve

## Abstract

**Background:**

Culture-negative endocarditis due to *Tropheryma whipplei* is a rare disease. Mostly the diagnosis is made by histologic examination of resected heart valve tissue.

**Case presentation:**

In this case report, we described a patient with a classical Whipple’s disease. Transesophageal echocardiography (TEE) showed a vegetation on noncoronary cusp of the aortic valve. Whipple’s disease was confirmed by positive *Tropheryma whipplei* polymerase chain reaction (PCR) in EDTA blood and a duodenal biopsy with positive periodic acid-Schiff stain (PAS) macrophages.

**Conclusion:**

Due to timely diagnosis, our patient was treated with antibiotics without valve replacement.

## Background

Endocarditis is a serious infectious disease. In some cases the blood cultures are negative. We described a patient with blood culture-negative endocarditis due to *Tropheryma whipplei*. Diagnosis is difficult to made, because fever and a history of valvulopathy of the Duke criteria are mostly absent [1]. The diagnosis is generally made with PCR or histologic examination of the resected heart valve tissue. In our case, diagnosis was made with TEE which showed a vegetation on noncoronary cusp of the aortic valve, positive PCR for *Tropheryma whipplei* in EDTA blood and a duodenal biopsy with positive PAS macrophages. Our patient was treated with antibiotics without valve replacement.

## Case presentation

A 52 year old male patient presented to the emergency room with an acute onset left sided hemiparesis. He complained of nausea and bowel discomfort for a long period of time and had lost weight. The patient had a history of alcohol and nicotine abuses and used to be homeless. He had periods of fever in the past months. He was regularly visiting a rheumatologist for artralgia of unknown origin. On physical examination we saw a thin, ill-kept Caucasian man with a blood pressure of 100/70 mmHg and a regular pulse of 80 beats/min and temperature of 36.5ºC. The heart sounds were normal and no murmurs were heard. Examination of the lungs and abdomen were normal and there were no stigmata of endocarditis. The electrocardiogram on admission showed sinus rhythm with complete left bundle branch block, chest X-ray was normal. Blood analysis showed a normocytic anaemia Hb 7.2 mmol/L, elevated C-reactive protein of 101 mg/L, thrombocytosis of 593/ml, white blood count of 5.9/nl, ASAT of 18/ U/l, ALAT of 8 U/l, serum creatinine of 54 mol/l with a MDRD GFR of >100 ml/min/1.73 m^2^.

**Figure 1:**
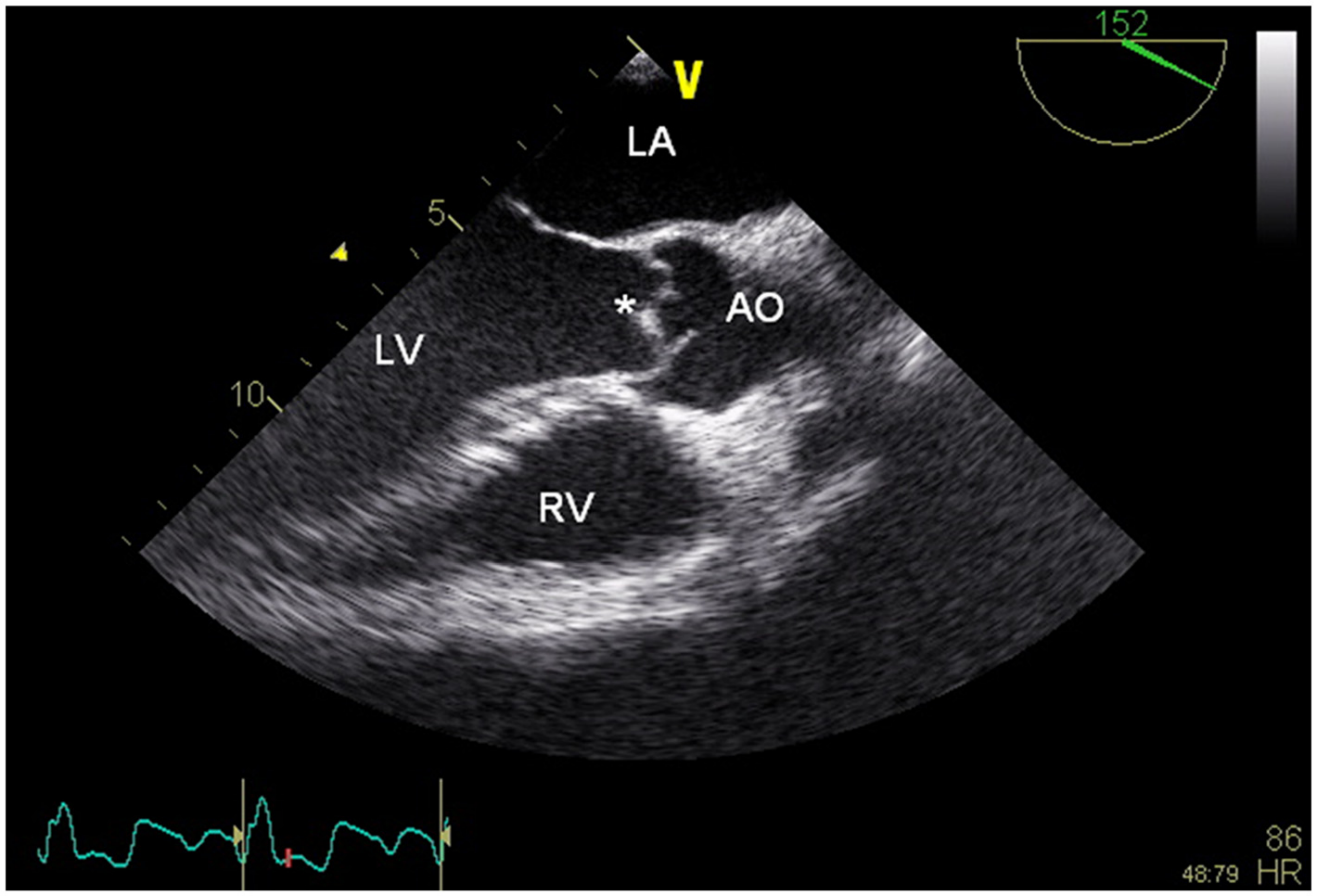
**Transesophageal echocardiography shows a vegetation on the noncoronary cups of the aortic valve.** LA: left atrium, LV: left ventricle, RV: right ventricle, AO: aorta, *:vegetation.

Computed tomography scan (CT scan) of cerebrum showed no bleeding or old infarction. An ischemic cerebral vascular accident was concluded. On TEE a vegetation on the noncoronary tricuspid cusp of the aortic valve was shown (Figure 1). Ventricles and atria were otherwise normal. We concluded that the patient had an endocarditis, most likely complicated by an embolus to the brain causing left sided hemiparesis.

**Figure 2:**
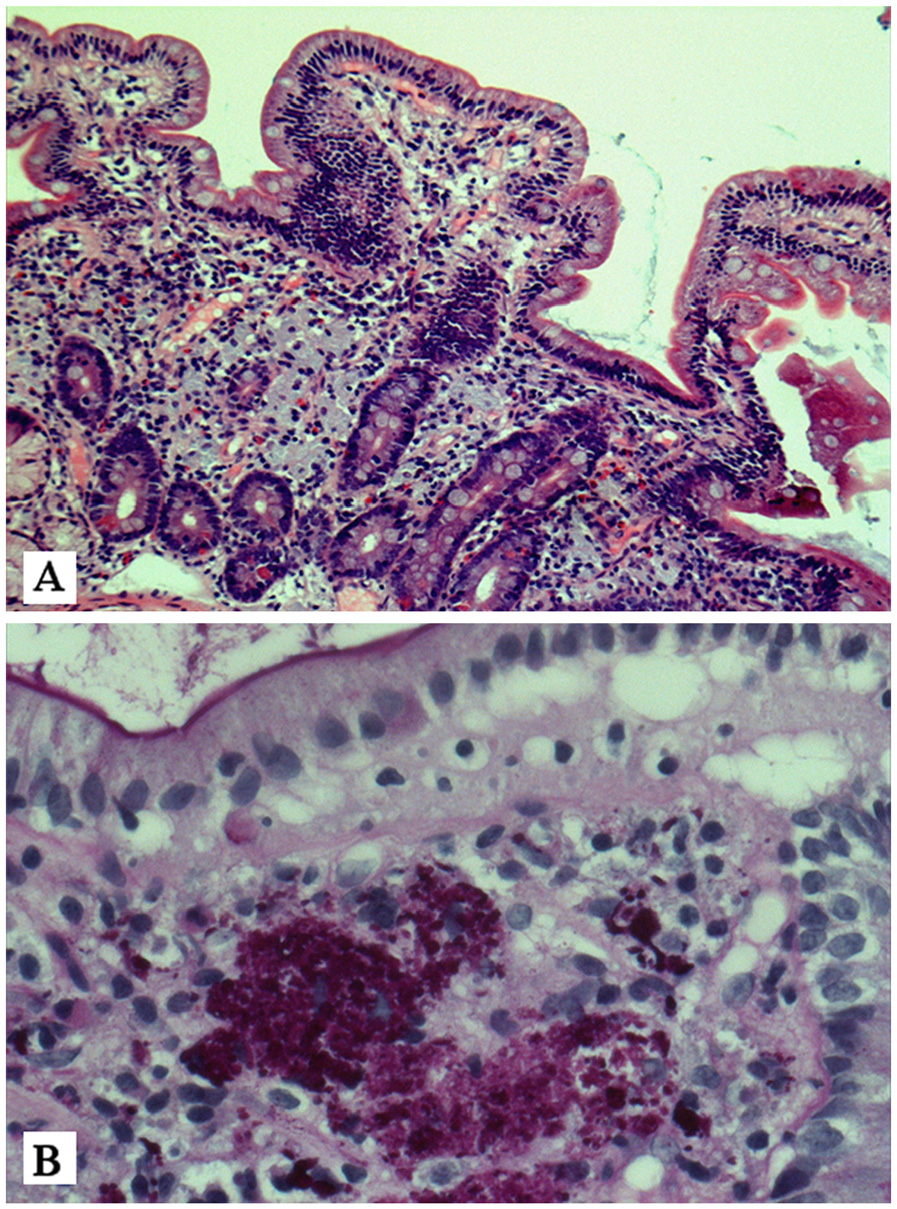
**A-B. Histological examination of duodenal mucosa, with histiocytes in the lamina propia (cytoplasma in light blue coloured) (hematoxylin stain, 10 x 40 magnification).** (**A**). Using PAS staining round structures are seen in the histiocytes, suggestive for Tropheryma whipplei (original magnification 40 x 40 magnification) (**B**).

Several blood cultures were drawn, but remained negative. Subsequently, other causes of endocarditis were investigated, like *Coxiella burnetii*, *Bartonella henselae* and *Tropheryma whipplei.* PCR for *Tropheryma whipplei* on EDTA blood was positive, where as *Coxiella burnetii* and *Bartonella henselae* PCR remained negative. With this in mind, combined with patients abdominal complaints duodenal biopsies were taken by gastroscopy. In the lamina propria, clusters of PAS-positive macrophages were detected, confirming the diagnosis of Whipple’s disease (Figure 2).

**Figure 3:**
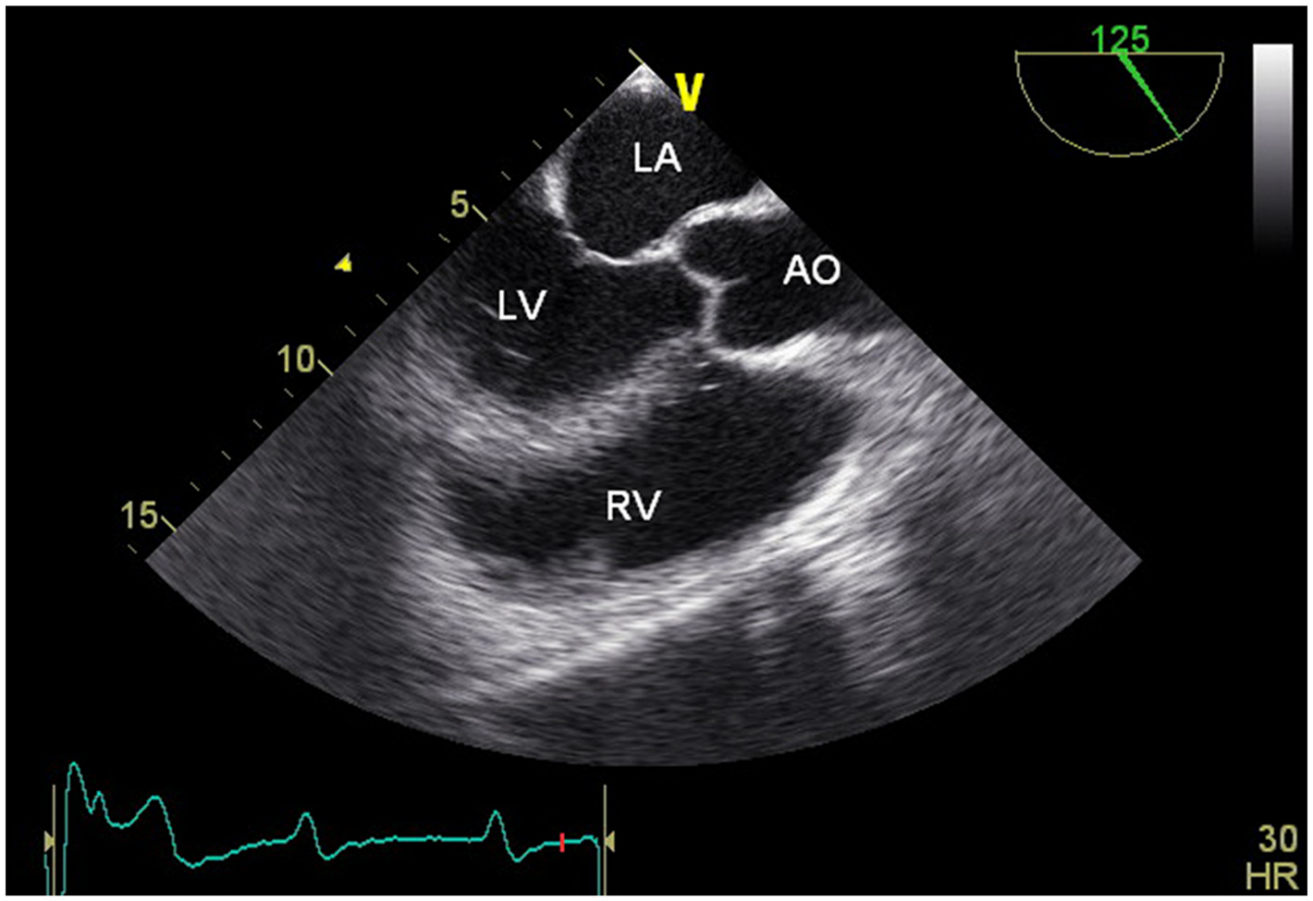
**Four weeks after treatment with penicillin intravenous transesophageal echocardiography shows no vegetations**. LA: left atrium, LV: left ventricle, RV: right ventricle, AO: aorta.

High doses of benzylpenicillin (12 ME/24 hours) was started initially, the hemiparesis fully recovered within a few days. On the sixth day of admission the patient developed asymptomatic Mobitz II atrioventricular block. A myocardial abscess was suspected but could not be confirmed by TEE or Magnetic Resonance Imaging (MRI). TEE showed a normal left ventricle function and mild aortic valve regurgitation, without residual vegetation (Figure 3). MRI of the heart showed no abscess. Four months after discharge, a symptomatic total atrioventricular block occurred and once again TEE showed no abscess. Patient received a permanent pacemaker.

During penicillin treatment, blood analysis showed an increase of C-reactive protein, whereafter trimethoprim-sulfametoxazole twice daily 960 milligram was added orally. *Tropheryma Whipplei* PCR was performed from EDTA blood two and three weeks after start of antibiotic treatment and was negative both times*.*

Patient was treated with benzylpenicillin intravenously for 4 weeks and with co-trimoxazole for 1 year. He was discharged in a good clinical condition. During treatment his gastrointestinal disorders and joint pains resolved completely. The vegetation on the aortic valve disappeared and infection parameters normalised. Two years after finishing antibiotic treatment he is in good clinical condition without signs of a relapse of Whipple’s disease.

Whipple’s disease was first described in 1907 by G.H. Whipple. The disease is extremely rare with an estimated incidence of below 1/1,000,000 [1]. Classical Whipple’s disease is characterised by gastrointestinal symptoms and arthralgias [2-4]. The main gastro intestinal symptoms are diarrhoea, weight loss and abdominal pain. Central nerve system (CNS) can be affected as well, leading mostly to headache and cognitive dysfunctions [5]. Severe complications of CNS infection, like insomnia and epilepsy are very rare. Hemiparesis could be due focal cerebral lesions, however we think that in our patient hemiparesis was probably due to an embolus of a vegetation. We also performed a MRI of the brain after 4 weeks of antibiotic treatment, this showed old infarction and no focal lesions as seen in central nervous system involvement by *Tropheryma whipplei*.

Most of the patients, about 85% are middle aged male, Caucasians are more affected then non-Caucasians [3,6]. The causative organism, *Tropheryma whipplei* is a gram-positive bacillus related to the group of Actinomycetes. It appears to be present in the environment, it has been found in sewage water and sewage plant workers [7,8]. About 66% of the patients had been exposed to animals or soil [8]. The pathogenesis of Whipple’s disease is unknown, although a lack of cell-mediated immunologic response is mentioned as predisposing factor in the literature [9].

The tropism of the bacillus is extensive and can affect several tissues like small-intestine colon, brain, heart, lung, kidney, bone marrow and skin [9]. Cardiovascular disease is present in up-to one-third of patients. Endocarditis is the most frequent presentation. Mitral and aortic valves are most often affected [3,10]. Diagnosis of Whipple’s disease, which is often delayed, is based on histological examination of duodenal biopsy and heart valves [4,10]. The presence of histiocytes in the lamina propia of the duodenal mucosa, containing periodic acid-Schiff (PAS) positive round structures are very suggestive for *Tropheryma whipplei*[4,10]. Immunohistochemistry can improve the identification. It is difficult to culture *Tropheryma whipplei*, detection of the bacterium is therefore based on DNA amplification techniques, like PCR [3]. Sensitivity of PCR from peripheral blood is good in advance cases, like our patient with endocarditis. According to the Duke criteria, confirmation of endocarditis with *Tropheryma whipplei* is not always possible. This, together with the rareness of this disease make under reportage very likely.

Progression of the infection is very slow. The outcome of patients with Whipple’s disease is good after an adequate antibiotic treatment. Penicilline G and third generation cefalosporines, like ceftriaxone, are recommended [4]. Especially when CNS is involved, these two antibiotics are given adequate high CSF levels. Trimehoprim-sulfamehoxazole (co-trimoxazol) is a good oral alternative and is mostly given, like in our patient, following primary intravenous therapy of penicilline or ceftriaxone [11]. Duration of treatment is variable, but most of the time lasting several months to a year. Relapse of Whipple’s disease can occur in 17-35% of patients, especially in patient not treated with penicillines [3,4].

In our case we were able to make a diagnosis without valve resection due to the combination of symptoms, TEE findings, the PCR result on peripheral blood and the PAS-positive macrophages in the duodenal biopsy. As far as we know, this is the third case described with *Tropheryma whipplei* endocarditis cured without valve replacement [12].

## Conclusion

Early detection, by PCR from peripheral blood has a positive effect on the outcome and can prevent surgical intervention. Therefore it is important to be aware of this disease in culture- negative endocarditis.

## Consent

Written informed consent was obtained from the patient for publication of this case report and any accompanying images. A copy of the written consent is available for review by the Editor-in-Chief of this journal.

## Competing interests

The authors declare that they have no competing interests.

## Authors’ contributions

All authors participated in design, coordination and helped to draft the manuscript. All authors read and approved the final manuscript.
